# Multiplexed Strain Phenotyping Defines Consequences of Genetic Diversity in Mycobacterium tuberculosis for Infection and Vaccination Outcomes

**DOI:** 10.1128/msystems.00110-22

**Published:** 2022-04-18

**Authors:** Allison F. Carey, Xin Wang, Nico Cicchetti, Caitlin N. Spaulding, Qingyun Liu, Forrest Hopkins, Jessica Brown, Jaimie Sixsmith, Rujapak Sutiwisesak, Samuel M. Behar, Thomas R. Ioerger, Sarah M. Fortune

**Affiliations:** a Department of Immunology & Infectious Diseases, Harvard T.H. Chan School of Public Health, Boston, Massachusetts, USA; b Division of Microbiology & Immunology, Department of Pathology, University of Utah, Salt Lake City, Utah, USA; c Immunology and Microbiology Program, Graduate School of Biomedical Science, University of Massachusetts Medical School, Worcester, Massachusetts, USA; d Department of Microbiology and Physiological Systems, University of Massachusetts Medical School, Worcester, Massachusetts, USA; e Department of Computer Science, Texas A&M University, College Station, Texas, USA; f Ragon Institute of MGH, MIT, and Harvard, Cambridge, Massachusetts, USA; University of California San Diego

**Keywords:** BCG, TnSeq, clinical strains, genomics, mycobacterium, tuberculosis, vaccination

## Abstract

There is growing evidence that genetic diversity in Mycobacterium tuberculosis, the causative agent of tuberculosis, contributes to the outcomes of infection and public health interventions, such as vaccination. Epidemiological studies suggest that among the phylogeographic lineages of M. tuberculosis, strains belonging to a sublineage of Lineage 2 (mL2) are associated with concerning clinical features, including hypervirulence, treatment failure, and vaccine escape. The global expansion and increasing prevalence of this sublineage has been attributed to the selective advantage conferred by these characteristics, yet confounding host and environmental factors make it difficult to identify the bacterial determinants driving these associations in human studies. Here, we developed a molecular barcoding strategy to facilitate high-throughput, experimental phenotyping of M. tuberculosis clinical isolates. This approach allowed us to characterize growth dynamics for a panel of genetically diverse M. tuberculosis strains during infection and after vaccination in the mouse model. We found that mL2 strains exhibit distinct growth dynamics *in vivo* and are resistant to the immune protection conferred by Bacillus Calmette-Guerin (BCG) vaccination. The latter finding corroborates epidemiological observations and demonstrates that mycobacterial features contribute to vaccine efficacy. To investigate the genetic and biological basis of mL2 strains’ distinctive phenotypes, we performed variant analysis, transcriptional studies, and genome-wide transposon sequencing. We identified functional genetic changes across multiple stress and host response pathways in a representative mL2 strain that are associated with variants in regulatory genes. These adaptive changes may underlie the distinct clinical characteristics and epidemiological success of this lineage.

**IMPORTANCE** Tuberculosis, caused by the bacterium Mycobacterium tuberculosis, is a remarkably heterogeneous disease, a feature that complicates clinical care and public health interventions. The contributions of pathogen genetic diversity to this heterogeneity are uncertain, in part due to the challenges of experimentally manipulating M. tuberculosis, a slow-growing, biosafety level 3 organism. To overcome these challenges, we applied a molecular barcoding strategy to a panel of M. tuberculosis clinical isolates. This novel application of barcoding permitted the high-throughput characterization of M. tuberculosis strain growth dynamics and vaccine resistance in the mouse model of infection. Integrating these results with genomic analyses, we uncover bacterial pathways that contribute to infection outcomes, suggesting targets for improved therapeutics and vaccines.

## INTRODUCTION

Pathogen population diversity can affect a range of clinically relevant phenotypes, including virulence, response to treatment, emergence of antibiotic resistance, and vaccine efficacy. In order to translate a basic understanding of pathogen biology into clinical advances and begin to move toward the goal of personalized medicine in infectious diseases, it is critical to assess the generalizability of a given observation to clinical pathogen populations. With the revolution in genome sequencing, we can envision a future in which the features of the pathogen are incorporated into medical decision making. Rapid, inexpensive sequencing technologies have transformed our ability to enumerate the genetic diversity within and between pathogen populations. Uncovering the consequences of these genetic variants for pathogen physiology and associating them with specific phenotypes has been most successful in the arena of antimicrobial resistance. This has been possible because drug resistance can be readily and reproducibly measured *in vitro*, and there are now widely used diagnostic assays that leverage the resulting genotype-phenotype associations to rapidly tailor antimicrobial regimens (1). However, many clinically relevant phenotypes, such as virulence, transmissibility, or likelihood of causing different disease manifestations, are less easily measured and may be confounded by variation in host features. In addition, we lack efficient experimental approaches to assess the functional consequences of pathogen genetic variation at scale and thus are limited in our capacity to create robust genotype-phenotype maps.

These challenges are particularly acute in the study of Mycobacterium tuberculosis, the etiologic agent of tuberculosis, which is a leading cause of infectious disease deaths worldwide (2). M. tuberculosis causes approximately 10 million active infections per year and is estimated to latently infect 1/4 of the world’s population (2). Whole-genome sequencing-based phylogenetic studies have demonstrated that M. tuberculosis strains segregate into seven distinct genetic lineages (Lineages 1 to 7) that have geographic origins reflecting evolution concurrent with early human migration (3, 4). Epidemiological studies have found associations between strain lineage and a range of clinical phenotypes, including disease progression, transmissibility, likelihood of antibiotic resistance, and the efficacy of vaccination (5–13). However, these associations are not always consistent from study to study (14) and are confounded by the strong geographic structure of the M. tuberculosis phylogeny, making the impact of pathogen variation difficult to distinguish from host and health system variation. Moreover, because manipulating M. tuberculosis is so cumbersome, the experimental characterization of strain differences has focused on a tiny number of reference strains; thus, it is often unclear whether the identified phenotypic characteristics are reflective of lineage-, sublineage-, or strain-level differences.

Several epidemiologic studies suggest that strains belonging to a sublineage of Lineage 2, the so-called “modern Beijing” lineage, here referred to as mL2, are associated with hypervirulence, increased transmissibility, treatment failure, and escape from the protection conferred by vaccination (5–13). Comparative phenotyping of an L4 reference strain, H37Rv, with an mL2 strain (HN878) demonstrated that mL2 strains synthesize phenolic glycolipid, a cell envelope lipid with immunomodulatory properties (15, 16), and that the associated polyketide synthase gene, *pks15-pks1*, is disrupted by a small deletion in L4 strains. Directed genetic studies of HN878 and H37Rv demonstrate that production of phenolic glycolipid increases virulence in mice, suggesting a model in which the increased virulence and transmission of mL2 strains compared to L4 strains can be at least partially attributed to this genetic difference. However, the presence of an intact *pks15-pks1* open reading frame does not strictly correlate with virulence across clinical isolates. Ancestral L2 strains and strains from Lineages 1 and 3, which are not associated with enhanced virulence, also possess an intact *pks15-pks1* gene (17–19).

The basis of other lineage-associated traits is even less well understood. mL2 strains are associated with the more frequent acquisition of multidrug resistance and treatment failure, and some mL2 strains have an increased basal mutation rate, leading to the hypothesis that there has been selection for the evolution of hypermutability to increase fitness in the setting of widespread antibiotic treatment (20, 21). These differences in mutability have been ascribed to sublineage-specific missense mutations in the DNA damage repair genes *mutT2*, *mutT4*, and *ogt* (22, 23). However, these variants have not been conclusively linked to hypermutability in experimental or observational studies (24–27). mL2 strains also possess genetic variants that result in the constitutive overexpression of the DosR regulon, a hypoxic response regulon hypothesized to confer a fitness advantage *in vivo* (28). However, DosR overexpression did not enhance M. tuberculosis fitness in an animal model of infection (28). Taken together, these data suggest that it is too simplistic to imagine that the complex clinical traits ascribed to different M. tuberculosis lineages are the result of any single mutation. Rather, the evolution of M. tuberculosis over time may have produced a network of interacting genetic variants resulting in the rewiring of key features of pathogen biology in a way that has modulated clinical characteristics. Consistent with this idea, a population genetic analysis of M. tuberculosis isolates found that nonsynonymous single nucleotide polymorphisms (SNPs) were overrepresented in transcriptional regulators in mL2 strains, a signature of selection and a potential mechanism for widespread functional genetic changes (29).

Ultimately, to incorporate bacterial features into the design and deployment of new diagnostics and treatments for M. tuberculosis, and in infectious diseases more generally, we need facile tools to rapidly phenotype clinical pathogen populations and to define the major molecular axes of biologic variation for traits beyond antimicrobial resistance. To address these limitations for M. tuberculosis, we demonstrate the feasibility of utilizing molecular barcoding to define strain and lineage growth dynamics in the mouse model of infection. Working from the hypothesis that infection phenotypes are likely multigenic, we employ a coordinated set of systems genomic tools to map the molecular basis of lineage-specific growth traits. We show that a representative mL2 strain exhibits broad rewiring of stress and host response pathways associated with variants in key regulatory genes. These adaptations may underlie this lineage’s unique clinical characteristics and global epidemiological success and reveals vulnerabilities that could be exploited to develop improved therapeutics and more effective vaccines.

## RESULTS

### Molecular barcoding of M. tuberculosis clinical isolates permits multiplexed phenotyping *in vitro* and *in vivo*.

**FIG 1:**
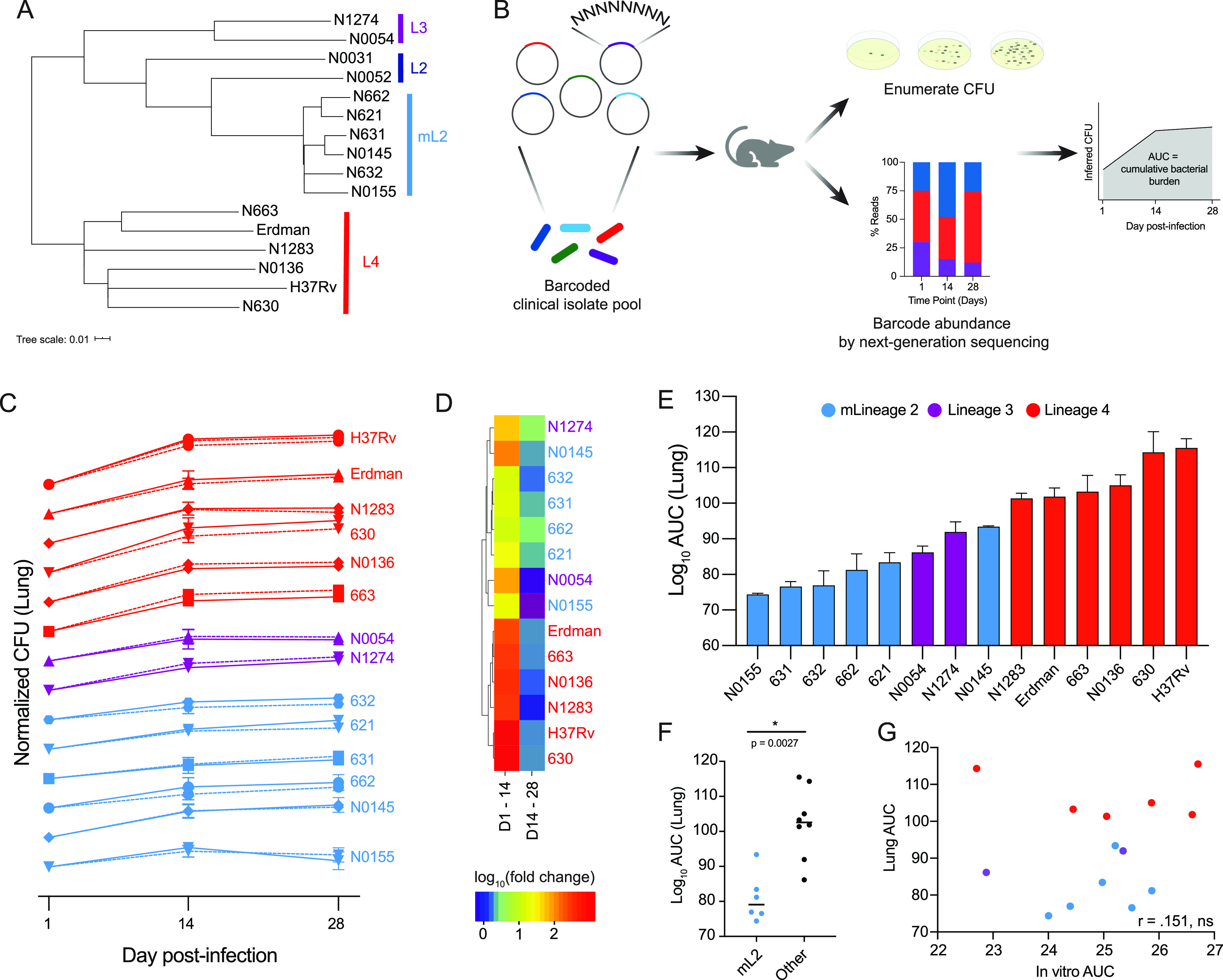
Barcoded pool of M. tuberculosis clinical isolates for multiplexed phenotyping. (A) Phylogenetic tree of M. tuberculosis isolates used in this study; an approximate maximum likelihood tree was generated with FastTree. (B) Strategy for barcoding and pooling isolates, performing mouse infections, calculating CFU, and determining cumulative bacterial growth. (C) Growth dynamics of M. tuberculosis isolates in the lung over the course of infection. Each strain’s CFU values were normalized to day 1 postinfection and log_10_ transformed. Data represent means with standard deviations (SD) (*n* = 4). Barcode replicates are shown as solid/dashed lines. (D) Hierarchical cluster analysis of strain growth rates over the first 2 weeks of infection and the second 2 weeks of infection. (E) Cumulative growth of each strain in the lung over the 4-week infection. Data represent mean replicate barcodes for each strain and standard errors of the means (SEM). (F) Growth in the lung of mL2 strains compared to all other strains, with significance determined by Mann-Whitney U test. (G) Correlation between cumulative bacterial growth *in vitro* and *in vivo* in the lung (Pearson correlation coefficient of log_10_ transformed data).

We sought to develop methodology to facilitate quantitatively robust, facile phenotyping of M. tuberculosis clinical strains. We previously demonstrated the utility of genetic barcoding to tag individual bacteria and isogenic strains in a population, which can then be assayed in experiments where competitive fitness is tracked through deep sequencing (30). We therefore prototyped a similar strategy to rapidly define the *in vivo* characteristics of a panel of M. tuberculosis clinical isolates. We assembled a panel of 16 clinical isolates, representing three epidemiologically prevalent lineages (L2, L3, and L4), and the widely used reference strains H37Rv and Erdman, which belong to L4 (Fig. 1A) (31, 32). Of the eight L2 strains in our panel, six belong to the “modern” Beijing sublineage, one belongs to the “ancestral” Beijing sublineage (N0052), and one belongs to the “proto” Beijing sublineage (N0031). We tagged each strain with a unique, 8-bp barcode that can be read out by next-generation amplicon sequencing (Fig. 1B). To provide an internal assessment of experimental reproducibility, each strain was barcoded in duplicate.

We then evaluated the viability of this approach to enumerate strain fitness *in vitro* and in an infection model. To measure *in vitro* growth dynamics, barcoded strains were pooled at equal ratios based on the optical density at 600 nm (OD_600_) and inoculated into standard medium. Bacteria were plated for CFU enumeration and genomic DNA extraction on days zero, three, and seven postinoculation. Barcode abundance was determined by amplicon sequencing (see Materials and Methods), and an inferred CFU value for each strain was calculated from the total number of CFU and relative barcode abundance at each time point (Fig. 1B). Inferred CFU values were normalized to input values. We found that growth rates of barcode replicates for each strain were highly correlated within experiments and across independent experiments (see Fig. S1A and B and Table S1 in the supplemental material).

10.1128/msystems.00110-22.1 FIG S1 (A) Growth dynamics of barcoded M. tuberculosis isolates in 7H9 medium. Each strain’s inferred CFU values were normalized to input and log_10_ transformed. Data represent means with SD (*n* = 3). Barcode replicates are shown as solid/dashed lines. (B) Correlation between bacterial growth rates in independent *in vitro* experiments (Pearson correlation coefficient of log_10_ transformed data). (C and D) Growth dynamics of barcoded M. tuberculosis isolates in C57BL/6 mice in lung (C) and spleen (D) tissue, including the ancestral and proto-Beijing strains N0052 and N0031. Each strain’s inferred CFU values were normalized to input and log_10_ transformed. Data represent means with SD (*n* = 4). Barcode replicates are shown as solid/dashed lines. (E) Correlation between bacterial growth rates in the lung in independent C57BL/6 mouse experiments (Pearson correlation coefficient of log_10_ transformed data). (F) Cumulative growth of each strain in the spleen over the four-week infection. Data represent mean replicate barcodes for each strain and SEM. (G) Growth in the spleen of mL2 strains compared to all other strains, with significance determined by Mann-Whitney U test. (H) Growth *in vitro* of mL2 strains compared to all other strains, with significance determined by Mann-Whitney U test. (I) Correlation between bacterial growth *in vitro* in 7H9 medium and *in vivo* in the spleen of C57BL/6 mice (Pearson correlation coefficient of log_10_ transformed data). Download FIG S1, TIF file, 2.6 MB. Copyright © 2022 Carey et al. 2022 Carey et al. https://creativecommons.org/licenses/by/4.0/ This content is distributed under the terms of the Creative Commons Attribution 4.0 International license.

Having demonstrated the capacity of this approach to robustly track bacterial strain growth dynamics *in vitro*, the barcoded pool was then used to infect C57BL/6 mice. One, 14, and 28 days postinfection, mice were sacrificed and spleen and lung tissue harvested for CFU enumeration and barcode abundance as described above. Each strain’s inferred CFU values were normalized to day one values. We found that growth rates of strain barcode replicates were highly correlated in both lung and spleen tissue (Fig. S1C and D, Table S1). We performed a second infection and found that strain growth rates in two independent experiments were also highly correlated (Fig. S1E). These results demonstrate that our barcoding approach permits highly reproducible, multiplexed tracking of M. tuberculosis growth dynamics over the course of infection.

10.1128/msystems.00110-22.6 TABLE S1 Barcode reproducibility. Pearson correlation coefficients for strain barcode replicates calculated from normalized, log_10_ transformed inferred CFU of *in vitro*, spleen, and lung data. The H37Rv correlation coefficient represents the average of the three pairwise barcode comparisons. Download Table S1, XLSX file, 0.01 MB. Copyright © 2022 Carey et al. 2022 Carey et al. https://creativecommons.org/licenses/by/4.0/ This content is distributed under the terms of the Creative Commons Attribution 4.0 International license.

### Barcoding reveals lineage-specific growth dynamics during infection.

Bacterial growth *in vivo* is an essential component of pathogenicity, and different growth rates may be advantageous during different disease stages and states. M. tuberculosis growth dynamics are characterized by an initial phase of relatively unchecked growth before an effective immune response can be mounted (33). This is followed by an extended, sometimes lifelong, period of reduced bacterial burden that represents the outcome of a dynamic interplay between pathogen growth and host-mediated killing (33). Some, but not all, animal studies have observed an increased bacterial burden among mL2 strains during acute infection, a trait that is suggested to provide a selective advantage (34–36).

Therefore, we sought to define strain and lineage growth dynamics during infection with our barcoding approach. We focused on mL2, L3, and L4, where we had several representative isolates, enabling us to draw more general conclusions about lineage traits. Because the lung is the physiological niche to which M. tuberculosis is adapted, we focused on bacterial growth phenotypes in this tissue, where we observed variable growth dynamics that appeared similar among strains of the same lineage (Fig. 1C). Hierarchical cluster analysis of growth rates confirmed that strains belonging to mL2 grouped together, while strains belonging to L4 grouped together (Fig. 1D). The growth dynamics of L4 were characterized by rapid growth over the first 2 weeks of infection, followed by a plateau over the second 2 weeks of infection. mL2 growth dynamics were characterized by slower growth over the first 2 weeks of infection and continued, steady growth over the following 2 weeks (Fig. 1C and D). Strains from L3 exhibited mixed growth dynamics.

We next assessed cumulative bacterial growth over the course of the infection by calculating the area under the curve (AUC) of the log-transformed, normalized CFU values (Fig. 1B). Unexpectedly, we found that bacterial growth in the lungs over the 4-week infection period was significantly less in the mL2 strains than in other strains (*P* = 0.0027) (Fig. 1E and F). Analysis of the spleen CFU data did not reveal a statistically significant growth difference between mL2 and other strains (*P* = 0.3450) (Fig. S1F and G) and mL2 strains are not universally slow growing, as they did not exhibit reduced growth *in vitro* in 7H9, a standard culture medium (*P* = 0.8518) (Fig. S1H). There was no correlation between cumulative bacterial growth under this *in vitro* condition and *in vivo* growth (Fig. 1G and Fig. S1I), suggesting that strain growth dynamics are sculpted by the host environment. To further test this hypothesis, we infected RAG1 knockout (KO) mice, which do not have mature B or T cells, with the barcoded pool. In this immunocompromised host background, we found that mL2 strains’ growth was not significantly less than that of other strains (*P* = 0.1419) (Fig. S2A). Taken together, these observations suggest that the slow growth of mL2 strains is in response to an intact immune response in the lung environment and not a global feature.

10.1128/msystems.00110-22.2 FIG S2 (A) Growth of mL2 strains compared to all other strains in the lung over two weeks in C57BL/6 mice (left) and RAG1 KO mice (right), with significance determined by Mann-Whitney U test. (B) Difference in bacterial burden in the spleen of C57BL/6 mice conferred by BCG vaccination over the course of the four week infection for each barcoded strain. Data represent mean replicate barcodes and SEM. (C) Protection conferred by BCG vaccination against mL2 strains compared to other strains in the spleen. Significance determined by Mann-Whitney U test. Download FIG S2, TIF file, 1.5 MB. Copyright © 2022 Carey et al. 2022 Carey et al. https://creativecommons.org/licenses/by/4.0/ This content is distributed under the terms of the Creative Commons Attribution 4.0 International license.

### BCG confers less protection against infection by mL2 strains.

The mL2 growth characteristics were surprising given our assumption that increased epidemiologic fitness would correlate with increased bacterial burden *in vivo*. However, more nuanced models for the increasing prevalence of mL2 suggest that this lineage has become epidemiologically dominant in the setting of widespread vaccination with Bacillus Calmette-Guerin (BCG) (37). BCG is a live, attenuated strain of Mycobacterium bovis whose protective efficacy is both incomplete and variable (38). One contribution to the variable efficacy of BCG is thought to be M. tuberculosis strain diversity, and some, but not all, studies have found that BCG has reduced efficacy against infection by mL2 strains (8, 36, 39–42). However, this has been difficult to assess in human population studies due to host and environmental confounders. Therefore, we next aimed to use our molecular barcoding approach to determine whether BCG confers equal protection against mL2 strains compared to strains from other lineages.

**FIG 2:**
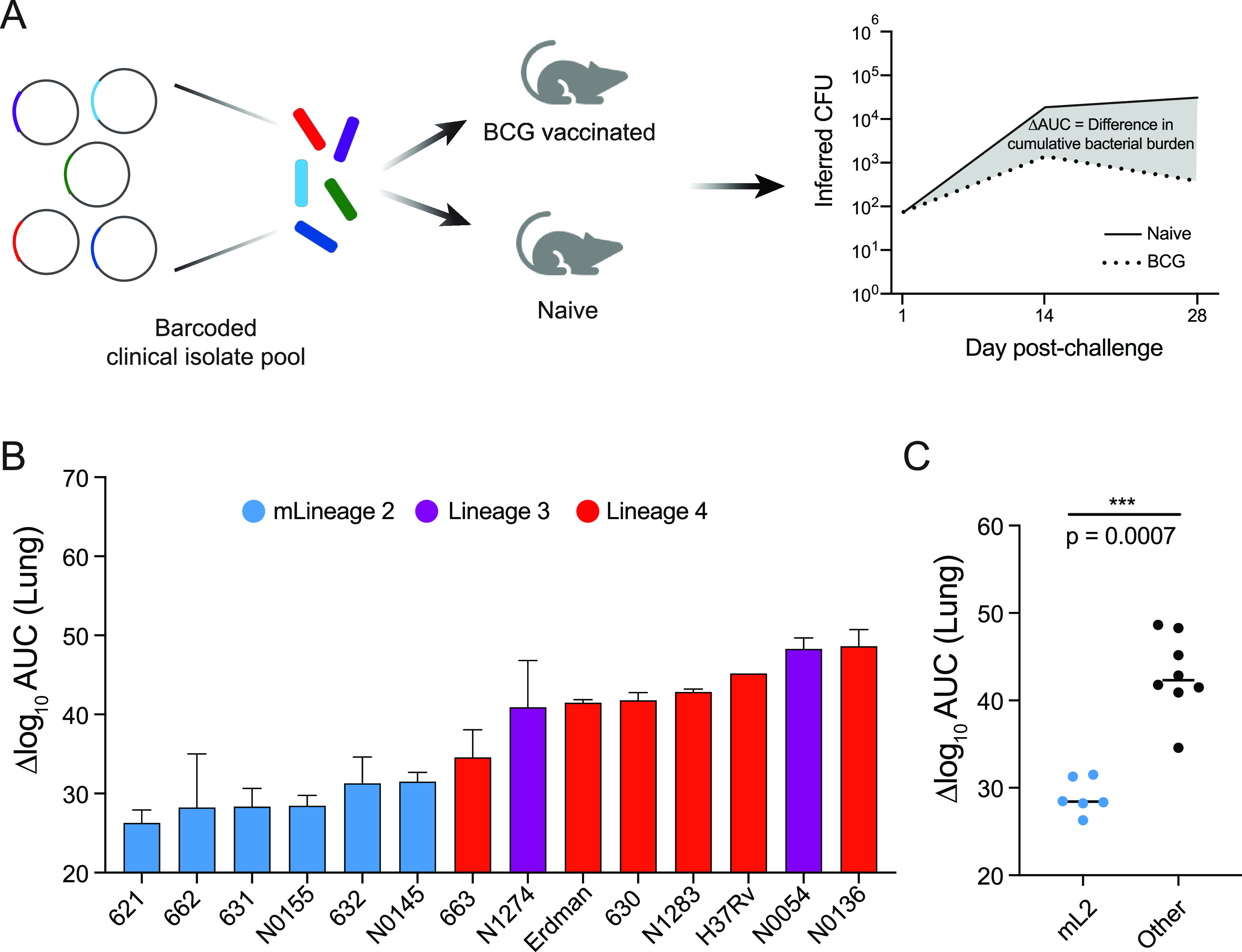
Defining strain and lineage contributions to BCG vaccine escape. (A) Strategy for vaccinating and challenging mice and quantifying protection. (B) Difference in bacterial burden in the lung conferred by BCG vaccination over the course of the 4-week infection. Data represent mean replicate barcodes and SEM. (C) Protection conferred by BCG vaccination against mL2 strains compared to all other strains, with significance determined by Mann-Whitney U test.

Mice were vaccinated subcutaneously with BCG, rested for 12 weeks to allow an adaptive immune response to develop, and then challenged with the barcoded M. tuberculosis pool (Fig. 2A). One day, 2 weeks, and 4 weeks postchallenge, lung and spleen tissue were harvested and CFU values inferred as described above. To quantify protection, we calculated the difference in cumulative bacterial growth over time between naive and BCG-vaccinated animals (Δlog_10_AUC). We found that the protection conferred by BCG vaccination varied by strain (Fig. 2B). Consistent with epidemiologic predictions, BCG conferred significantly less protection against mL2 strains than other strains in the pool in lung tissue (*P* = 0.0007) (Fig. 2C). The protection conferred by BCG was tissue specific, and there was no difference in protection between mL2 strains and other strains in the spleen (Fig. S2B and C).

### Strain-specific differences in gene expression under stress conditions.

Together, these data indicate that mL2 strains have *in vivo* traits that are not neatly classified as hypervirulence. To better understand the relevance of these features to the more complex context of human infection, we sought to identify bacterial pathways shaping the *in vivo* biology of mL2 strains. Comparative genomic and population genetic analyses have identified a number of sequence variants specific to mL2 strains and found that variants in regulatory genes are overrepresented (22, 29). These genetic changes include nonsynonymous SNPs in the *dosR-dosS-dosT* and *kdpD-kdpE* two-component systems, the serine/threonine protein kinase *pknA*, the LuxR family regulators *Rv0890c* and *Rv2488c*, and the tetR family regulators *Rv0452* and *Rv0302*, among others. The impact of most of these variants for pathogenesis has not been determined; however, this sequence-level analysis suggests differential engagement of key regulatory nodes at the host-pathogen interface in mL2 strains, with potential consequences for infection phenotypes.

**FIG 3:**
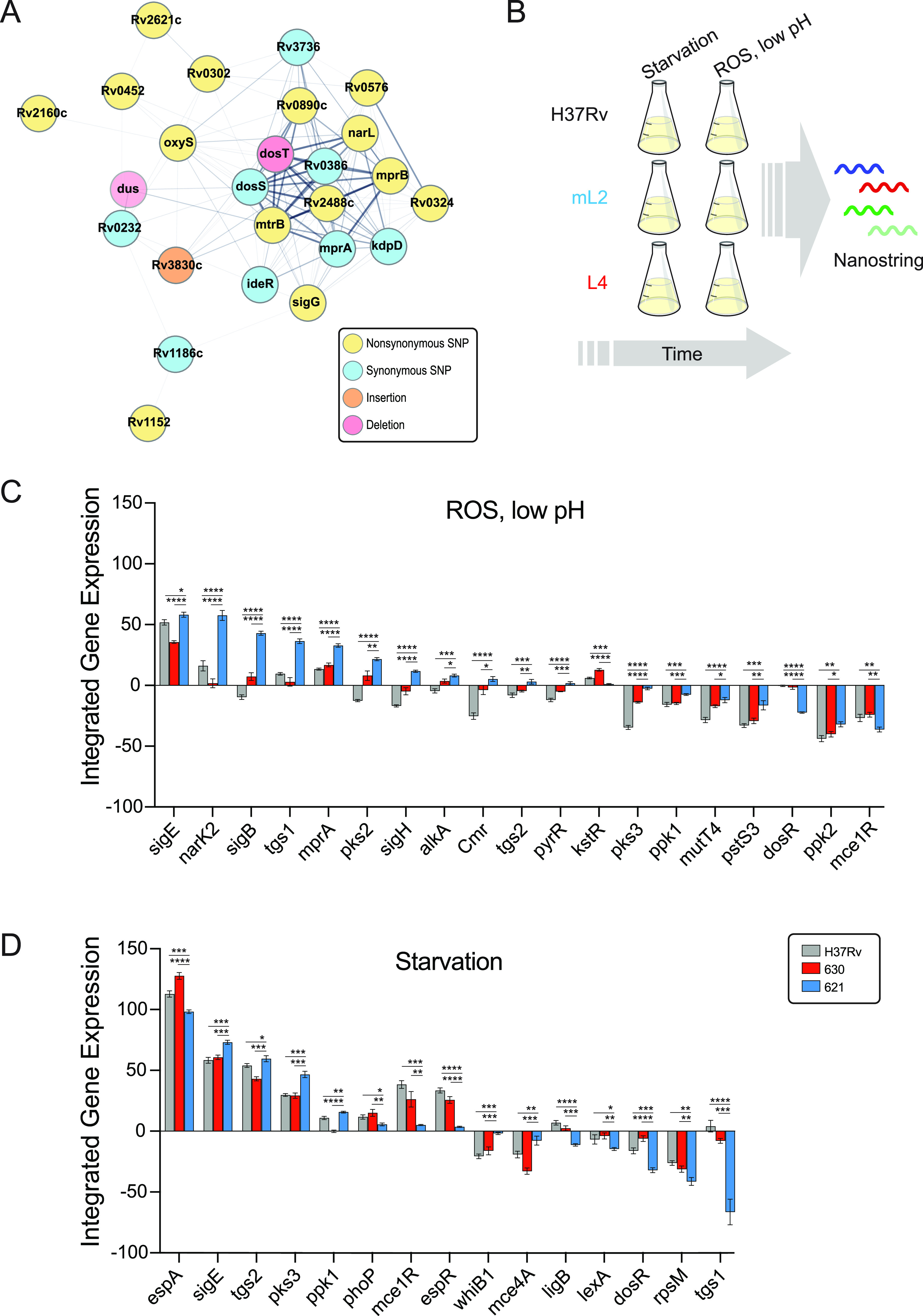
Transcriptional signatures under stress conditions differ between M. tuberculosis strains. (A) STRING plot of regulatory genes with coding region variants specifically in the mL2 strain 621 compared to the L4 strain 630 and the reference strain H37Rv. Edge thickness represents strength of evidence for direct interaction. (B) Experimental strategy for the *in vitro* stress gene expression experiment. (C and D) Genes with quantitative and qualitative differences in expression in the mL2 strain under oxidative stress, low-pH conditions (C), and starvation conditions (D) over the course of the experiment. Asterisks indicate significant differences in integrated gene expression over time, determined by calculating the area under the curve for T0 normalized, log_2_ transformed data and performing one-way ANOVA with Tukey’s posttest for significance.

To test this model, we selected representative mL2 (621) and L4 (630) strains from the barcoded panel in addition to the widely used reference strain, H37Rv, which belongs to L4, for further characterization. We included a clinical isolate from L4 as a comparator because it is likely that H37Rv has adaptations due to continuous laboratory culture (43). First, we identified genetic variants specific to the mL2 strain 621 compared to H37Rv and the L4 clinical isolate (Table S2). Consistent with published studies, we identified variants in regulatory genes, including a 1-bp deletion in the gene encoding the DosT sensor kinase, which has been linked to overexpression of the DosR hypoxia-responsive regulon under exponential growth conditions (44) as well as synonymous and nonsynonymous SNPs in the genes encoding the MprA/B two-component system, which regulates numerous stress and host response pathways, including alternative sigma factors and the ESX-1 virulence system (Fig. 3A) (45, 46).

10.1128/msystems.00110-22.7 TABLE S2 Genetic variants in mL2 strain 621. Download Table S2, XLSX file, 0.08 MB. Copyright © 2022 Carey et al. 2022 Carey et al. https://creativecommons.org/licenses/by/4.0/ This content is distributed under the terms of the Creative Commons Attribution 4.0 International license.

Given the large number of genetic changes we identified through this sequence analysis, we reasoned that rather than reductionist, single-variant studies, we would employ systems biology approaches to gain insight into the molecular basis of mL2 strain infection phenotypes. Because we identified a number of genetic differences in critical regulators of bacterial adaptation to host-imposed stresses, we decided to assess the transcriptional responses of these strains under *in vitro* conditions that mimic the phagolysosomal environment inhabited by M. tuberculosis, specifically, oxidative stress at low pH and nutrient starvation (Fig. 3B). To do so, we designed a custom NanoString probe set to measure expression of 54 curated bacterial stress regulators and downstream response genes (Table S3). These targets were selected because they have been shown to be induced during infection or under *in vitro* conditions that approximate the infectious milieu (47–50). RNA was extracted 2, 6, and 24 h after stress induction, and reads were normalized to internal controls and time point 0 (T0; see Materials and Methods). Hierarchical cluster analysis revealed concerted changes in gene expression under each condition, consistent with prior reports (Fig. S3) (47). Because we measured gene expression at multiple time points, we integrated normalized NanoString counts over time for a more robust assessment of each strain’s transcriptional response. To identify lineage-specific differences in expression, we filtered for genes that were both quantitatively and qualitatively differentially expressed in the mL2 strain compared to both H37Rv and the L4 clinical isolate (Fig. 3C and D and Table S3).

10.1128/msystems.00110-22.3 FIG S3 Heatmaps of NanoString gene expression for H37Rv, 621, and 630 strains under oxidative stress and low pH (A) and starvation (B). Each gene’s counts were normalized to input (T0) values and expressed as log_2_(fold change). Download FIG S3, TIF file, 2.1 MB. Copyright © 2022 Carey et al. 2022 Carey et al. https://creativecommons.org/licenses/by/4.0/ This content is distributed under the terms of the Creative Commons Attribution 4.0 International license.

10.1128/msystems.00110-22.8 TABLE S3 NanoString target sequences and gene expression data. Target sequences for gene expression experiments. NanoString counts were normalized to internal control and housekeeping probes for each of the three replicates at T0 and each time point under the *in vitro* stress conditions, and average AUC values and standard errors were derived from log_2_(fold change) data normalized to the T0 values for each strain. Significance was determined by one-way ANOVA. Download Table S3, XLSX file, 0.05 MB. Copyright © 2022 Carey et al. 2022 Carey et al. https://creativecommons.org/licenses/by/4.0/ This content is distributed under the terms of the Creative Commons Attribution 4.0 International license.

Among this set of differentially expressed genes, we observed higher expression of the alternative sigma factors *sigB*, *sigE*, and *sigH* as well as the two-component sensor *mprA* under the low-pH, oxidative stress condition (Fig. 3C) and higher expression of *sigE* under starvation (Fig. 3D). *sigE* is considered a master regulator of mycobacterial gene expression under stress conditions (51), while *sigB* appears to be an end regulator in the sigma factor cascade (52). *sigE*, *sigB*, and *sigH* are part of a transcriptional circuit with the MprA/B two-component system, a central sensor of environmental stresses and key determinant of mycobacterial persistence during infection (46, 53–55).

Previous studies have found that *dosR* expression is constitutively higher in mL2 strains (28, 44), which we also observed in the T0 data (Table S3); however, we found that *dosR* expression was significantly lower in the mL2 strain under both stress conditions (Fig. 3C and D). This suggests that the mL2-specific *dosR* genetic variants alter the transcriptional response of this regulator under stress conditions as well as under basal conditions, potentially in diverging ways. A subset of the *dosR* regulon genes was included in our expression panel: *narK2* (nitrate transport) and *tgs1* (triacylglycerol synthase). Both genes were differentially expressed in the mL2 strain under the tested stress conditions, displaying condition-specific expression profiles, with higher expression of *narK2* and *tgs1* under the low-pH, oxidative stress condition and decreased expression of *tgs1* under starvation. This likely reflects the integration of signals from multiple regulators to generate a response appropriate for both gene function and environmental conditions. Taken together, these targeted expression data indicate that mL2 strains have a distinct transcriptional response to the stresses experienced during infection that may impact virulence traits.

### Functional genomic analysis of M. tuberculosis strains during infection.

An alternative to using whole-genome sequencing and expression analyses to develop models of the biological pathways driving pathogen phenotypes is instead to leverage a functional genomic method: transposon sequencing (TnSeq). TnSeq entails genome-wide transposon mutagenesis coupled with next-generation sequencing and is a high-throughput, unbiased approach to defining bacterial genetic requirements for survival and growth under a condition of interest (56). In contrast to sequence analyses, where the biological consequences of individual variants may be difficult to predict, or transcriptomics, which can discount the role of constitutively expressed genes and posttranscriptional regulation, TnSeq provides a functional readout of the fitness cost of gene disruption. Importantly, strain-to-strain differences in genetic requirements identified by TnSeq have been shown to reflect meaningful differences in bacterial physiology (32, 57, 58). Therefore, we sought to use this approach to comprehensively define functional genetic differences in mL2 strains during infection.

To do so, C57BL/6 mice were infected with saturated transposon libraries of the three strains subjected to sequence and expression analyses: mL2 strain 621, L4 strain 630, and reference strain H37Rv. Because we observed the greatest distinctions in bacterial growth dynamics between mL2 and other strains 2 weeks postinfection (Fig. 1D), we chose 1- and 2-week time points for TnSeq analysis. We initially sought to compare genetic requirements during infection among strains by performing comparisons of each strain’s *in vitro* and *in vivo* libraries using a permutation test-based method to identify genes with statistical differences in read count (Table S4). Using this approach, we identified 137 genes that were essential for infection in all three of the M. tuberculosis isolates at either time point and as many as 132 genes required specifically in one strain. Among the core essentials are genes involved in critical metabolic processes (*bioA*), cell wall processes (*pbpA*), and nutrient acquisition (*mbtA*). However, a limitation of a binary classification system is that quantitative differences in genetic requirements are not uncovered. For example, a gene might be classified as nonessential for infection in all strains, yet the relative fitness cost of disrupting the gene may differ and can reflect important physiological differences among strains (32, 59).

10.1128/msystems.00110-22.9 TABLE S4 TnSeq transit resampling, genetic interactions, and HMM output. Transit analyses was performed as detailed in Materials and Methods, and .wig input data were generated by the TPP module of Transit from transposon junction sequencing data. Download Table S4, XLSX file, 18.2 MB. Copyright © 2022 Carey et al. 2022 Carey et al. https://creativecommons.org/licenses/by/4.0/ This content is distributed under the terms of the Creative Commons Attribution 4.0 International license.

**FIG 4:**
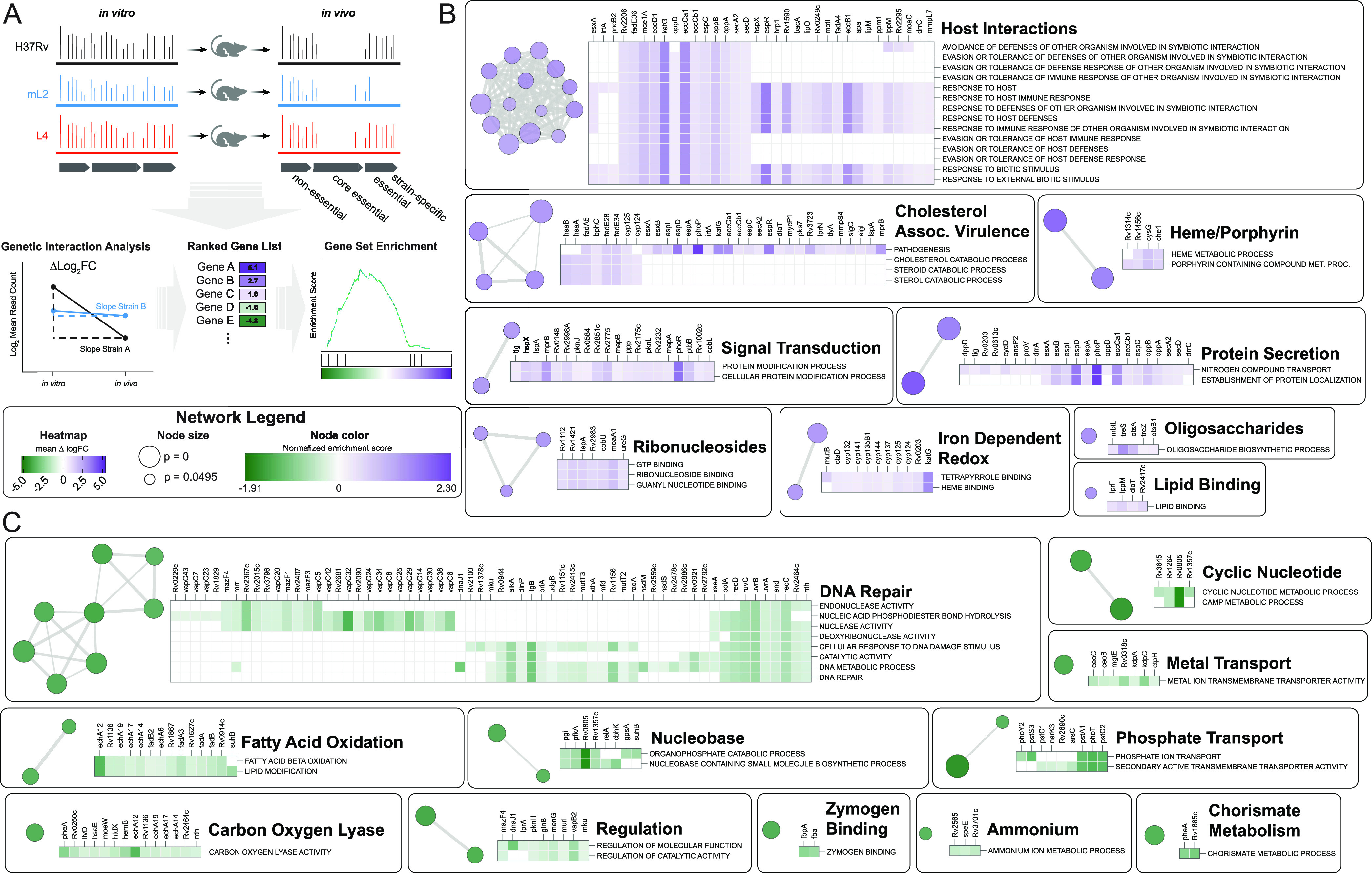
Functional genomics to identify genetic determinants of mL2 infection phenotypes. (A) Experimental strategy and analytic approach to defining differences in relative genetic requirements between strains during infection using transposon sequencing and genetic interactions analysis. (B and C) Network plots generated in Cytoscape depicting genes that have a decreased requirement (B) in the mL2 strain compared to the reference strain, H37Rv, 1 week postinfection or an increased requirement (C) by GSEA. Nodes represent enriched Gene Ontology (GO) terms with a cutoff of *P* < 0.05. GO terms that were also significant in the comparison between H37Rv and the L4 clinical isolate 630 were excluded. Node color represents normalized enrichment score. Node size is inversely proportional to significance value. Edge thickness represents the number of overlapping genes, determined by the similarity coefficient. Heatmaps display leading edge genes for each cluster, with color corresponding to the Δlog_2_(fold change) values of the genetic interactions TnSeq analysis.

Capturing such quantitative differences from a conditional TnSeq data set requires accounting for differences in the input libraries that exist due to both the stochastic nature of transposon mutagenesis and biological differences among strains. To accomplish this, we applied a Bayesian method that performs a four-way comparison of transposon-junction read counts across input and output libraries and compares the relative change in transposon mutant abundance (Fig. 4A) (60). This interaction analysis identifies genes that are conditionally essential *in vivo* in a strain-dependent manner. This pipeline was originally developed to identify epistatic genetic interactions between deletion strain and wild-type backgrounds; however, we reasoned that it could be used to identify differences in genetic requirements between strains of distinct genetic backgrounds.

We therefore performed pairwise interaction analysis between the reference strain H37Rv and each of the clinical isolates at each time point (Table S4). To define differences in genetic requirements specific to the 621 clinical isolate during infection, we considered only genes that were statistically significant (*P* value of <0.05) in the H37Rv-621 comparison but not significant in the H37Rv-630 comparison. By these criteria, 32 genes were differentially required in the mL2 strain 1 week postinfection, and 118 genes were differentially required 2 weeks postinfection. These gene sets were highly overlapping, as 21 of the 32 genes significant at week one were significant 2 weeks postinfection. To gain insight into the biological processes that differ among strains during infection, we performed gene set enrichment analysis (GSEA) on the output of the interaction analysis, using the Δlog_2_(fold change) values as input for the preranked method and Gene Ontology (GO) terms for functional annotation (Fig. 4A) (61). GSEA found that compared to the reference strain H37Rv, the mL2 isolate had 73 significantly enriched GO terms (*P* < 0.05). To identify pathways that were enriched specifically in the mL2 strain, 25 GO terms that were significant in the comparison between H37Rv and 630 were excluded. The remaining 48 GO terms indicated a decreased requirement in the mL2 strain for genes involved in host interactions, including the canonical virulence system, ESX-1; cholesterol catabolism; protein secretion; and heme metabolism (Fig. 4B and Table S5). There was an increased requirement in the mL2 strain for genes involved in DNA damage repair, phosphate uptake, fatty acid oxidation, and cyclic nucleotide signaling, among others (Fig. 4C). We found similar differences in GO term enrichment when comparing the mL2 and L4 clinical isolates head to head (Table S5), indicating that the observed differences do not simply reflect laboratory adaptation of H37Rv. Most of these processes were also enriched at the 2-week time point (Fig. S4), suggesting sustained, strain-specific differences in host-pathogen interactions during infection.

10.1128/msystems.00110-22.4 FIG S4 (A) Network plots generated in Cytoscape depicting GSEA of genes with differential requirements in the mL2 strain compared to the reference strain, H37Rv, two weeks postinfection. Nodes represent enriched Gene Ontology (GO) terms with a cutoff of *P* < 0.05. GO terms that were also significant in the comparison between H37Rv and the L4 clinical isolate 630 were excluded. Node color represents normalized enrichment score. Node size is inversely proportional to significance value. Edge thickness represents the number of overlapping genes, determined by the similarity coefficient. Heatmaps display leading edge genes for each cluster, with color corresponding to the Δlog_2_(fold-change) values of the genetic interaction TnSeq analysis. (B) Line plots showing log_2_(fold-change) trajectories over the course of the two-week infection for leading edge genes of selected functional groupings found to be enriched by GSEA of the TnSeq data (H37Rv v. mL2 strain 621). Thin lines represent individual genes, and thick lines represent the average for each functional grouping. Download FIG S4, TIF file, 2.2 MB. Copyright © 2022 Carey et al. 2022 Carey et al. https://creativecommons.org/licenses/by/4.0/ This content is distributed under the terms of the Creative Commons Attribution 4.0 International license.

10.1128/msystems.00110-22.10 TABLE S5 GSEA analysis of TnSeq data. Output of gene set enrichment analysis using the preranked method, with the transit genetic interaction Δlog_2_(fold change) values used as input. Download Table S5, XLSX file, 0.4 MB. Copyright © 2022 Carey et al. 2022 Carey et al. https://creativecommons.org/licenses/by/4.0/ This content is distributed under the terms of the Creative Commons Attribution 4.0 International license.

To place the variability in genetic requirements we observed between bacterial isolates from different phylogenetic lineages into broader biological context, we considered a recently published TnSeq study that investigated M. tuberculosis requirements for infection across genetically and immunologically diverse mouse backgrounds (62). In this study, an H37Rv transposon library was used to infect a panel of 60 mouse genotypes encompassing strains from the Collaborative Cross Collection and mice with specific immunological deficits, such as gamma interferon (IFN-γ) knockout. This approach facilitated a comprehensive assessment of variation in bacterial genetic requirements under distinct infection conditions. Consistent with our work and previous studies, the authors identified 234 genes required for H37Rv to grow or survive in C57BL/6 mice, yet there were as many as 212 additional *in vivo*-essential genes per mouse genotype. This is comparable to the 155 genes we identified as differentially required to infect C57BL/6 mice in the mL2 isolate compared to H37Rv, suggesting that the functional genetic differences between M. tuberculosis strains can be as substantial as those that are imposed by distinct host backgrounds. Through network analysis, the authors found that differentially required genes could be clustered into 20 modules with correlated changes in fitness. We performed a statistical analysis of the overlap between these modules and the genes that were differentially required in the mL2 strain during infection and identified three modules with significant overlap (*P*-adj. < 0.05, Fisher’s exact test). These modules are categorized as ESX-1, phosphate uptake, and an uncategorized set that includes a number of DNA damage repair genes. This intersection of host and pathogen variability suggests that certain lineages of M. tuberculosis may be adapted to specific host environments, consistent with population genomic analyses (4).

### Regulatory variants are associated with differential genetic requirements during infection.

Our TnSeq data indicate widespread functional genetic differences between M. tuberculosis strains over the course of infection. We noticed that many of the GO terms found to be enriched by GSEA in the mL2 strain represent biological processes regulated by genes with 621-specific genetic variants. For example, cholesterol metabolism genes are differentially required in 621, and this strain possesses a SNP upstream of *kstR*, which controls the cholesterol catabolism regulon. This suggests that rewiring of the bacterial response to the host environment is driven by selection on regulatory genes, consistent with sequence analyses of mL2 genomes (22, 29).

**FIG 5:**
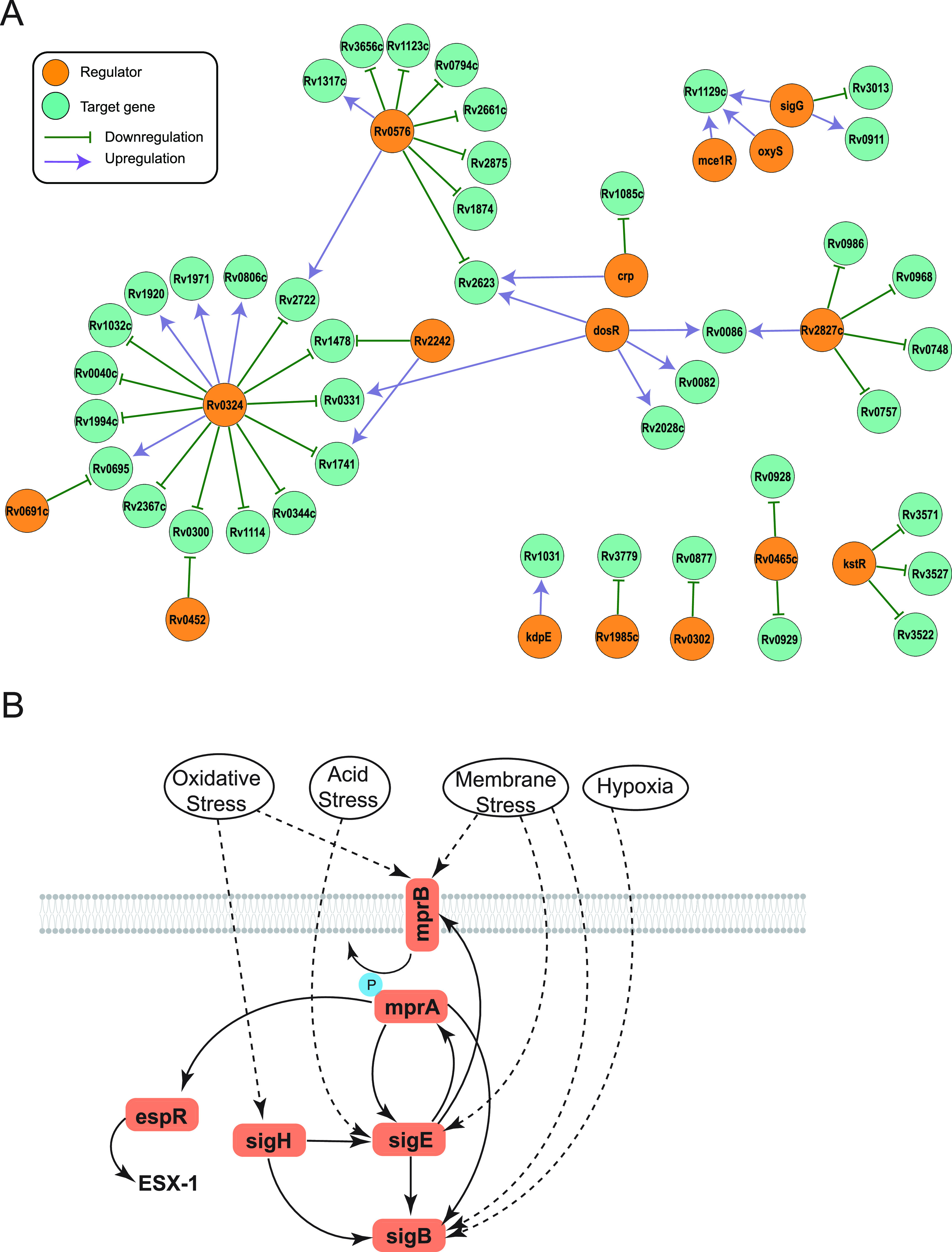
Differentially required genes are regulated by transcription factors with strain-specific variants. (A) Network plot generated in Cytoscape showing genes with mL2 strain-specific TnSeq differences that are transcriptionally regulated by systems with strain-specific genetic variants. (B) Schematic depicting the complex regulatory circuit of the two-component system MprA/B, which has an nsSNP in the sensor gene *mprB* in strain 621.

To test this hypothesis, we mined a published data set from a comprehensive M. tuberculosis transcription factor overexpression (TFOE) study (63). In this work, 206 of the 214 known and predicted M. tuberculosis transcription factors were inducibly overexpressed and transcriptional signatures assessed by high-density microarray, reflecting both direct and indirect regulatory effects. We integrated these data with our TnSeq results to determine which differentially required genes (as determined by genetic interactions analysis) were regulated by transcription factors with sequence variants. In cases such as the DosR regulon, where variants are located in the sensor of a two-component system, we considered genes regulated by the transcription factor. We found that 42 of the 129 genes that were differentially required specifically in mL2 strain 621 were regulated by a transcription factor possessing a 621-specific genetic variant (Fig. 5A). To assess the statistical significance of this finding, we performed a simulation with a null distribution of 10,000 trials of 129 genes chosen at random and found the overlap to be highly significant (*P* = 0.0048). This result is consistent with a model in which variants in response regulators drive functional genetic differences among strains.

This analysis likely underestimates the relationship between genetic variants in transcriptional regulators and the differential genetic requirements identified by TnSeq in this representative mL2 strain. In the TFOE study, transcriptional responses were assessed under a single, *in vitro* growth condition at a single time point, and stringent statistical thresholds were used to determine regulatory relationships. This may mask subtle but biologically important regulatory roles. For example, the transcriptional activator *mprA*, part of the MprA/B two-component system, was not found to regulate any genes by the rigorous thresholds of the TFOE study. However, directed genetic studies have found that *espR* is regulated by *mprA* (45, 64), and the sensor kinase of this system, *mprB*, has a nonsynonymous SNP in strain 621 (Fig. 3A and Table S2). *espR* regulates the ESX-1 virulence system, which was differentially required by the mL2 strain during infection (Fig. 4B and Table S4). MprA/B is also part of a regulatory loop with the alternative sigma factors *sigB*, *sigE*, and *sigH* (Fig. 5B); therefore, genetic variants at the top of this cascade may have pleotropic transcriptional effects.

These regulatory gene variants and associated functional genetic changes could confer resistance to the killing mechanisms imposed by the host or could reflect compensatory changes due to differences in the cellular microenvironment experienced by different strains. To distinguish between these possibilities, we performed a time-kill assay with the barcode library under low pH and nitrosative stress, conditions encountered by the bacterium during infection. We found that survival under acid stress was significantly higher for the mL2 strains than the other strains in the panel (Fig. S5A). There was no significant difference in survival under nitrosative stress; however, there was not substantial killing of either group at the dose used in this experiment (Fig. S5A). Taken together, these findings suggest that the functional genetic changes in mL2 strains are protective rather than compensatory.

10.1128/msystems.00110-22.5 FIG S5 (A) Bacterial survival at the indicated time points for mL2 strains compared to other strains in the barcode pool under low pH (left) and nitrosative stress (right). Each dot represents the mean fraction of surviving input bacteria, averaged across barcode replicates (*n* = 3). Significance determined by Mann-Whitney U test of log-transformed fractional survival. (B) Schematic of growth regulation in mL2 strains in response to host-induced stresses. Download FIG S5, TIF file, 0.9 MB. Copyright © 2022 Carey et al. 2022 Carey et al. https://creativecommons.org/licenses/by/4.0/ This content is distributed under the terms of the Creative Commons Attribution 4.0 International license.

## DISCUSSION

Tuberculosis is a notoriously heterogeneous disease, with outcomes ranging from lifelong, asymptomatic latency to primary progressive disease. Dissecting the contribution of bacterial genetic variation to this heterogeneity has been limited by confounding host and environmental factors in population studies and by the experimental intractability of M. tuberculosis in laboratory studies. Here, we developed a robust molecular barcoding approach that allowed us to characterize *in vivo* growth dynamics in a high-throughput fashion for a genetically diverse panel of M. tuberculosis isolates. Among these isolates are strains from the so-called “modern Beijing” sublineage (mL2), a sublineage that has been expanding in population size over the past 2 centuries, possibly due to traits that confer a selective advantage (65). One of the features attributed to mL2 strains in some epidemiological and small-animal studies is increased virulence (6, 8, 9, 13, 15). Therefore, it was unexpected that our *in vivo* fitness phenotyping revealed reduced cumulative bacterial growth of mL2 strains over the course of infection compared to other strains in our panel. An explanation for this discrepancy may be that many previous animal studies used a single strain or small number of strains isolated from outbreaks, such as HN878, that might inadvertently bias toward hypervirulence. In this study, we included mL2 strains from a reference set that was curated to be representative of each lineage (31). Thus, slower bacterial growth during acute infection may be more typical of the growth dynamics of mL2 than prior studies suggested. Indeed, M. tuberculosis is a pathogen that can infect an individual for a lifetime without a measurable increase in bacterial burden, and slow growth may be a survival strategy that circumvents immune-mediated killing (66). Therefore, perhaps it is not surprising that an epidemiologically successful lineage of M. tuberculosis exhibits reduced growth compared to other strains, at least during the early stages of infection.

Our barcoding approach also permitted a systematic examination of M. tuberculosis strain and lineage contributions to the efficacy of BCG vaccination, an unresolved question in the field. The importance of M. tuberculosis strain variation for vaccine efficacy has been difficult to assess in population studies, where host and environmental factors also vary. Our findings in the mouse, a relevant preclinical model for M. tuberculosis vaccination studies, experimentally confirm observations made in some epidemiological studies of reduced BCG efficacy against mL2 strains. This suggests that as new tuberculosis vaccines are designed, they should be evaluated for efficacy against genetically diverse and epidemiologically prevalent strains, and our barcoding approach provides a scalable means to do so.

Barcoding also makes assessing vaccine efficacy and pathogenesis traits in other infection models more efficient and experimentally tractable. Two mouse backgrounds were used in this work, including C57BL/6, which is widely used and recapitulates many features of human M. tuberculosis infection (67). However, there are important aspects of human disease, such as latency and transmission, that are not captured by this model yet may contribute to the epidemiologic success of the mL2 sublineage. Other mouse genotypes and other animal species more accurately reflect some aspects of human tuberculosis, and a key aim of future work is to define bacterial strain fitness and vaccine efficacy across diverse host backgrounds.

Together, these studies demonstrate the power of molecular barcoding for high-throughput phenotyping of bacterial strains, an approach that is applicable to numerous pathogens. While robust measurements of bacterial growth can be performed, bacterial burden is not the only feature driving virulence, and the multiplexed nature of this approach incurs limitations. These include the nonphysiologic intravenous route of infection required to prevent bottlenecking of the pool; the possibility that the immune response to some strains in the pool may impact the growth of other strains; and the inability to investigate immunopathology. While phenotypes that transcomplement will not be uncovered, this is a feature of other pooled phenotyping techniques, such as TnSeq and CRISPRi, which have nevertheless revealed important biological principles about numerous pathogens. For these reasons, single-strain infections will be an important next step in validating the findings made here. Despite these limitations, the lineage-level consistency in strain growth and vaccination phenotypes we observe reveal important features about M. tuberculosis biology that would be prohibitively onerous to uncover with a traditional, single-strain approach.

M. tuberculosis is an obligate human pathogen that is exquisitely adapted to the hostile environment of the lung and has evolved a suite of mechanisms to survive the stressors it encounters during infection (68). Therefore, we reasoned that it was unlikely that a single genetic change would be responsible for the clinical features attributed to mL2 strains or the phenotypes we observed in the mouse model. Thus, we elected to employ systems approaches that could interrogate coordinated, pathway-level changes in representative isolates. Our subsequent transcriptional and functional genomic studies indicate that the mL2 strain is functionally rewired across numerous stress and host response pathways. The genes we identified by TnSeq with mL2-specific differential requirements during infection represent key adaptive processes, including the ESX-1 virulence system, lipid metabolism, and DNA damage repair. A limitation of M. tuberculosis TnSeq studies is that due to the large number of mutants, selection must occur in the spleen to prevent bottlenecking of the transposon library. Therefore, an important goal of future work is to validate the genetic requirements identified by TnSeq in the aerosol infection model and to investigate how genetic requirements change over time, given the relatively short length (2 weeks) of our TnSeq experiments.

Our analysis indicates that the differentially required genes identified by TnSeq are more likely to be regulated by transcription factors with strain-specific variants than chance, a potential mechanism of evolutionary adaptation. Population genomic analyses are consistent with this observation, having found that transcriptional regulators are enriched for variants in mL2 (22, 29). Indeed, studies across other prokaryotic species suggest that evolution of transcription factor network structure is an important means of phylogenetic diversification and can lead to the emergence of organisms with distinct responses to environmental stimuli (69). What is the role of these variants for strain fitness during infection? The reduced cumulative growth of mL2 strains observed during infection could reflect increased bacterial killing by the host or could reflect growth regulation by the bacterium. Our *in vitro* kill curves suggest that mL2 strains are not more susceptible to killing by antibacterial mechanisms employed by the host, and our RAG1 KO data suggest that mL2 strains are capable of robust growth in the absence of an adaptive immune response. Therefore, our working model is that the reduced *in vivo* growth of mL2 strains in immunocompetent mice reflects regulated, protective growth slowing triggered by cues in the host environment (see Fig. S5B in the supplemental material). An important goal for future studies is to validate this model by testing the role of the mL2 genetic variants for transcriptional response networks and infection phenotypes.

A limitation of our transcriptional and functional genomic studies is that only one clinical isolate each from mL2 and L4 was characterized. The selected strains were representative of their lineage in growth characteristics and genetic features. However, in addition to lineage-level genetic diversity, strain-level genetic diversity has the potential to affect pathogenic traits. Variants present in some, but not all, strains within a lineage represent an evolutionary sandbox for selection, and dissecting the consequences of both levels of genetic variation for bacterial fitness can help define the selective landscape shaping M. tuberculosis’s ongoing adaptation. Such studies are now feasible with barcoding, which can facilitate phenotyping of numerous strains at scale under a range of *in vitro* and *in vivo* conditions. Coupled with computational techniques such as bacterial genome-wide association, the pathogen genes and variants that drive infection outcomes and response to clinical interventions such as vaccination can be uncovered, leading to the development of molecular diagnostics to guide more effective clinical care.

## MATERIALS AND METHODS

### Bacterial strains.

Clinical strains were identified as previously described and cultured from single colonies (4, 31). Strains were grown at 37°C and cultured in Middlebrook 7H9 salts supplemented with 10% oleic acid-albumin-dextrose-catalase (OADC), 0.5% glycerol, and 0.05% Tween 80 or plated on 7H10 agar supplemented with 10% OADC, 0.5% glycerol, and 0.05% Tween 80 unless otherwise noted. Clinical strains were handled to minimize *in vitro* passaging. Strains were previously whole genome sequenced as described previously (31, 32). To compare genomic variants between H37Rv, mL2 strain 621, and L4 strain 630, a custom assembly and variant-calling pipeline were used as previously described (32).

### Animals.

Female C57BL/6 mice were purchased from Jackson Laboratories (Bar Harbor, ME.). Mice were 6 to 8 weeks old at the start of all experiments, and infected mice were housed in biosafety level 3 (BSL3) facilities under specific-pathogen-free conditions at HSPH. The protocols, personnel, and animal use were approved and monitored by the Harvard University Institutional Animal Care and Use Committee.

Male and female RAG1 KO mice were 8 to 12 weeks old at the start of experiments, and infected mice were housed in BSL3 facilities under specific-pathogen-free conditions at UMMS. The protocols, personnel, and animal use were approved and monitored by the UMMS Institutional Animal Care and Use Committee.

### BCG vaccination.

Bacillus Calmette-Guerin, originally obtained from Statens Serum Institute, was prepared as previously described (70). Mice were immunized with 100 μL of frozen bacterial culture (OD_600_, 1.0; 2e7 CFU) subcutaneously in the left flank. Mice were rested for 12 weeks postvaccination prior to challenge.

### Barcoded clinical isolate growth *in vitro*.

M. tuberculosis strains were tagged with a random 8-bp barcode essentially as described previously (30). Single colonies of each strain were picked and Sanger sequenced to identify the barcode; colonies with two unique barcodes for each strain were selected. Barcoded strains were grown to log phase, pooled based on OD_600_ at approximately equal ratios, and frozen into aliquots. An aliquot was subsequently inoculated into 7H9 medium, grown to mid-log phase, and then back diluted to an OD_600_ of 0.01 in 7H9 in triplicate and incubated with shaking at 37°C. At the indicated time points, an aliquot was removed from each replicate for CFU enumeration, and an aliquot was removed for plating to recover ∼5e3 CFU as estimated by OD_600_ of the culture. Recovered CFU were scraped for genomic DNA extraction, amplicon Illumina sequencing, and barcode abundance quantification by custom Python scripts, essentially as described previously (30).

### Barcoded clinical isolate growth under *in vitro* stress conditions.

An aliquot of the frozen barcoded strain pool was inoculated into 7H9 medium, grown to mid-log phase, and then back diluted to an OD_600_ of 0.01 in freshly made stress medium or control medium in triplicate. Control medium consisted of 7H9 supplemented with 10% OADC, 0.5% glycerol, and 0.05% tyloxapol. For nitrosative stress, DETA-NO (Sigma-Aldrich) was added to the control medium at a final concentration of 1 mM. For acid stress, control medium was buffered to pH 4.5 with hydrochloric acid. An aliquot from each replicate was plated 5 days postinoculation for CFU enumeration and to recover ∼5e3 CFU for processing as described above.

### Barcoded clinical isolate mouse infections and analysis.

An aliquot of the barcoded strain pool was used for tail vein infection at 1e6 CFU/mouse. At indicated time points postinfection, spleens and lungs were harvested, homogenized, and plated on 7H10 supplemented with glycerol, Tween, OADC, and 20 μg/mL kanamycin. After 3 weeks of incubation, CFU were enumerated and 1e4 CFU were scraped for genomic DNA extraction, amplicon Illumina sequencing, and barcode abundance quantification by custom Python scripts, essentially as described previously (30).

### Gene expression.

For oxidative and starvation stress conditions, triplicate cultures of the indicated strains were grown to mid-log phase in 7H9, pelleted, washed once in an equal volume of Tris-buffered saline (TBS) supplemented with 0.05% tyloxapol, and then resuspended in freshly made stress medium as detailed below or 7H9 with 0.05% tyloxapol. For oxidative stress, bacteria were resuspended in 7H9 with 0.05% tyloxapol buffered to pH 4.5 with 10 μg/mL menadione. For starvation, bacteria were resuspended in TBS with 0.05% tyloxapol. Cultures were incubated at 37°C with shaking and aliquots removed for RNA extraction at the indicated time points. RNA was isolated essentially as described previously and quantified by Qubit RNA assay (Thermo Fisher) (32). A total of 125 ng of RNA was used as input in a NanoString assay with a custom-designed probe set (NanoString Technologies). Target sequences are listed in Table S3. Data were analyzed with nSolver version 4 (NanoString Technologies); raw NanoString counts were normalized to internal positive controls to correct for technical variation between assays and normalized to housekeeping genes (*ansA*, *aceAa*, and *secA2*) to correct for variation in RNA input (Table S3). Normalized counts were expressed as log_2_ (fold change) relative to T0, and data clustering was performed in R v4.0.3 using complete linkage and Euclidean distance. For statistical comparisons between strains, AUC of the log_2_(fold change) expression data over time were calculated and one-way analysis of variance (ANOVA) with Tukey’s posttest performed in R v4.0.3 (Table S3).

### Transposon library mouse infections and analysis.

Mice were infected via tail vein injection with 2e6 CFU of frozen aliquots of previously generated H37Rv or clinical strain *Himar1* transposon libraries (32). At the indicated time points postinfection, spleens were harvested, homogenized, and plated on 7H10 supplemented with glycerol, Tween, OADC, 0.2% Casamino acids (Difco), and 20 μg/mL kanamycin. For each mouse, 1e6 surviving colonies were scraped after 3 weeks for genomic DNA extraction and transposon-junction sequencing essentially as previously described (32). Reads were mapped to the H37Rv genome, and statistical comparisons of read counts between conditions and strains were performed using Transit v3.2.0 (71). To compare input (*in vitro*) and output (*in vivo*) libraries from each strain, the Transit resampling method was used, with insertions in the central 90% of each open reading frame considered and a locally estimated scatterplot smoothing (LOESS) correction for genome positional bias. To identify differences in genetic requirements during infection between strains, the Transit genetic interactions (GI) method was used (60). Gene set enrichment analysis and leading edge analysis were performed on the Transit GI-generated Δlog_2_(fold change) values using the GSEA v4.1.0 preranked tool (61). Genes classified as essential for *in vitro* growth in at least two of the three isolates were excluded from GSEA (Table S4). To identify *in vitro* genetic requirements for each strain, the Transit hidden Markov model (HMM) method was used, with insertions in the central 90% of each open reading frame considered and a LOESS correction for genome positional bias (72). Repetitive regions, deleted genes, and genes in a large duplicated region in the mL2 strain 621 were excluded as previously described (Table S4) (32).

### Data availability.

All relevant data to support the findings of this study are located within the paper and supplemental material. Software used in this study, as detailed above, included R v4.0.3 (https://www.r-project.org), Transit v3.2.0 (https://orca1.tamu.edu/essentiality/transit/), Cytoscape v3.8.2 (https://cytoscape.org), and GSEA v4.2.1 (https://www.gsea-msigdb.org/gsea/index.jsp).
