# Supplementary figures and images for: Multiplexed Strain Phenotyping Defines Consequences of Genetic Diversity in Mycobacterium tuberculosis for Infection and Vaccination Outcomes
Source: mSystems. 2022 Apr 18;7(3):e00110-22. doi: 10.1128/msystems.00110-22 (PMC9239107; doi:10.1128/msystems.00110-22)

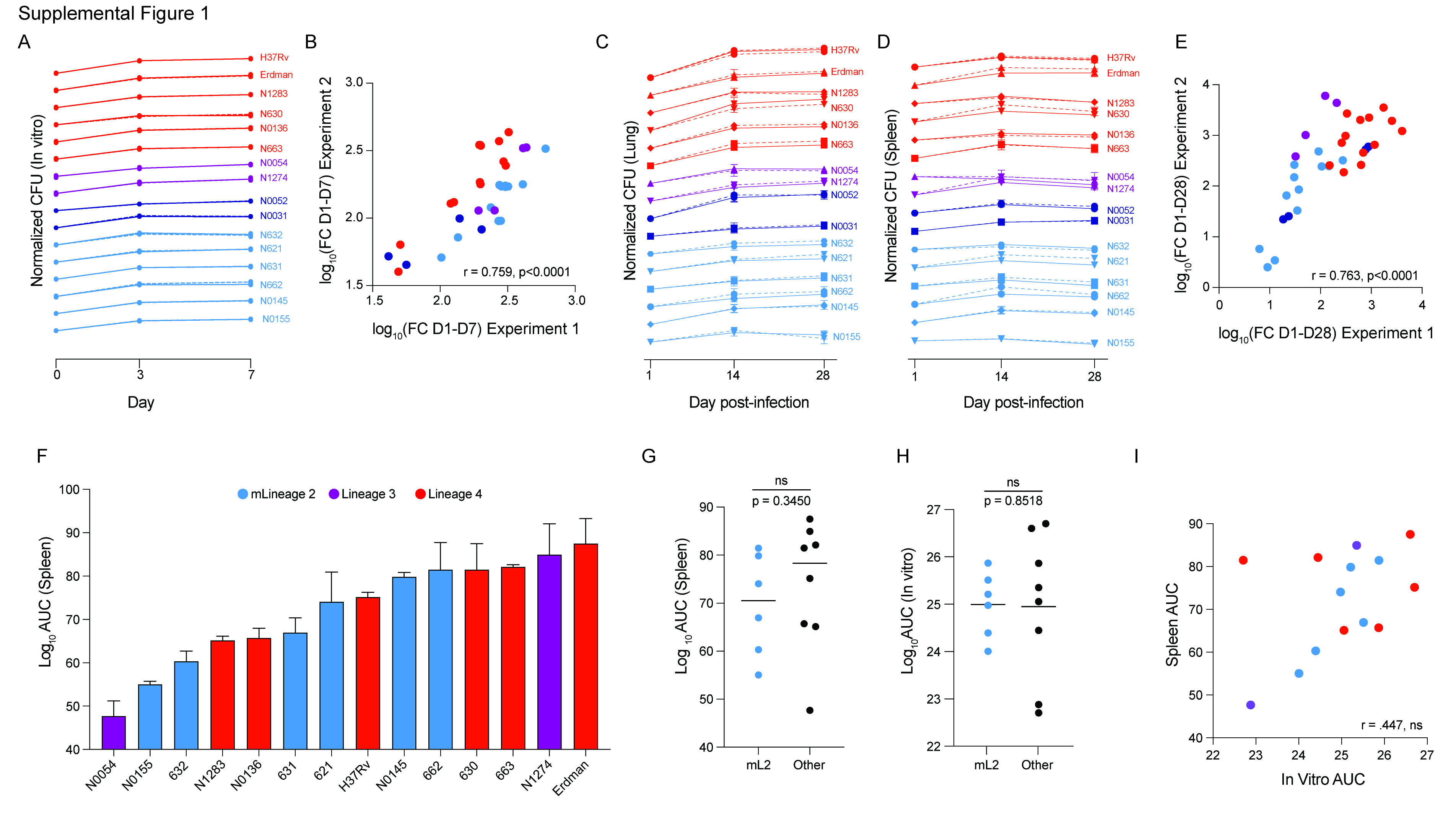

Supplement: FIG S1 [file msystems.00110-22-s0001.tif]

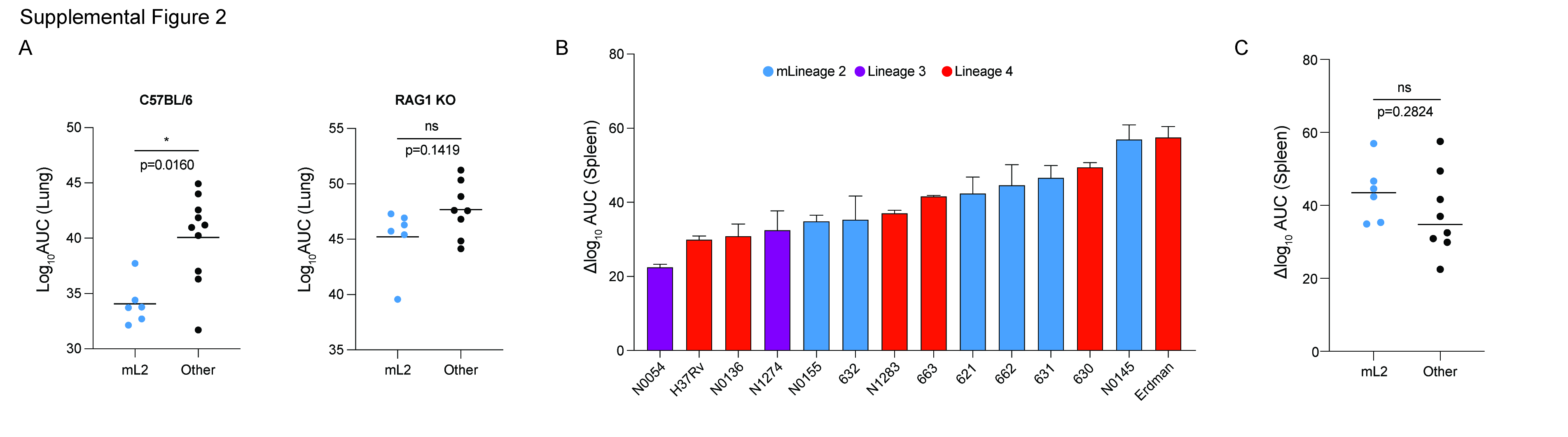

Supplement: FIG S2 [file msystems.00110-22-s0002.tif]

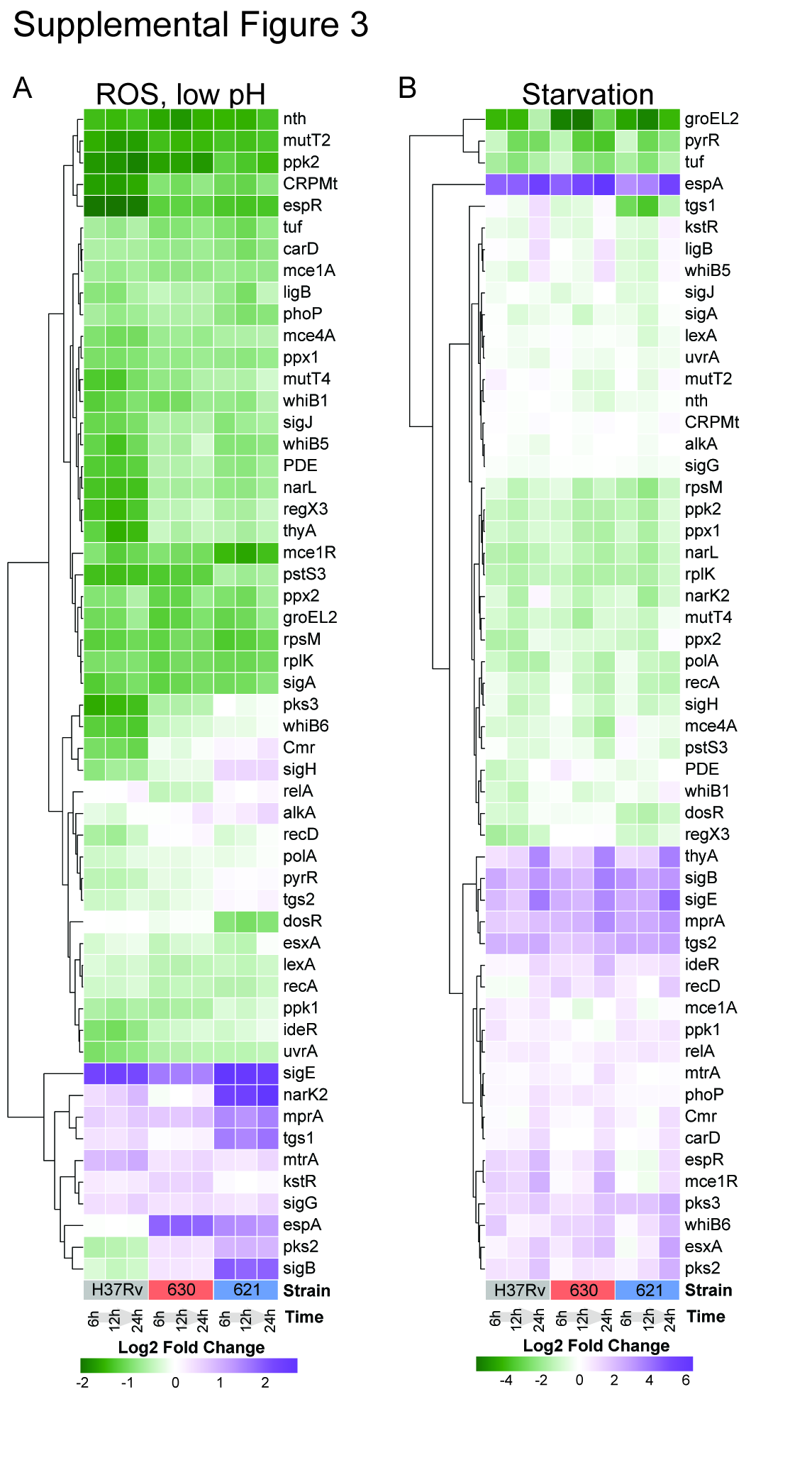

Supplement: FIG S3 [file msystems.00110-22-s0003.tif]

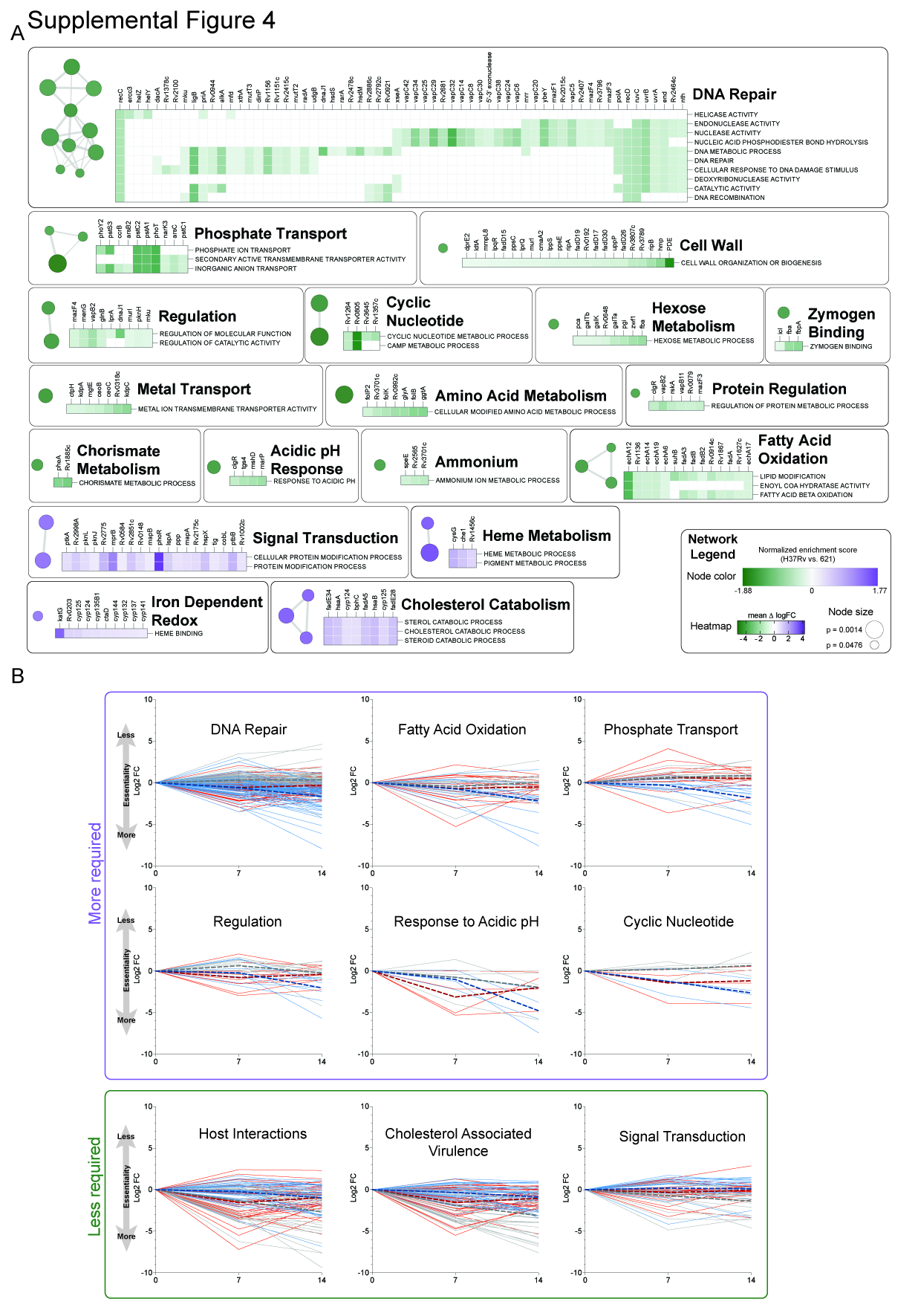

Supplement: FIG S4 [file msystems.00110-22-s0004.tif]

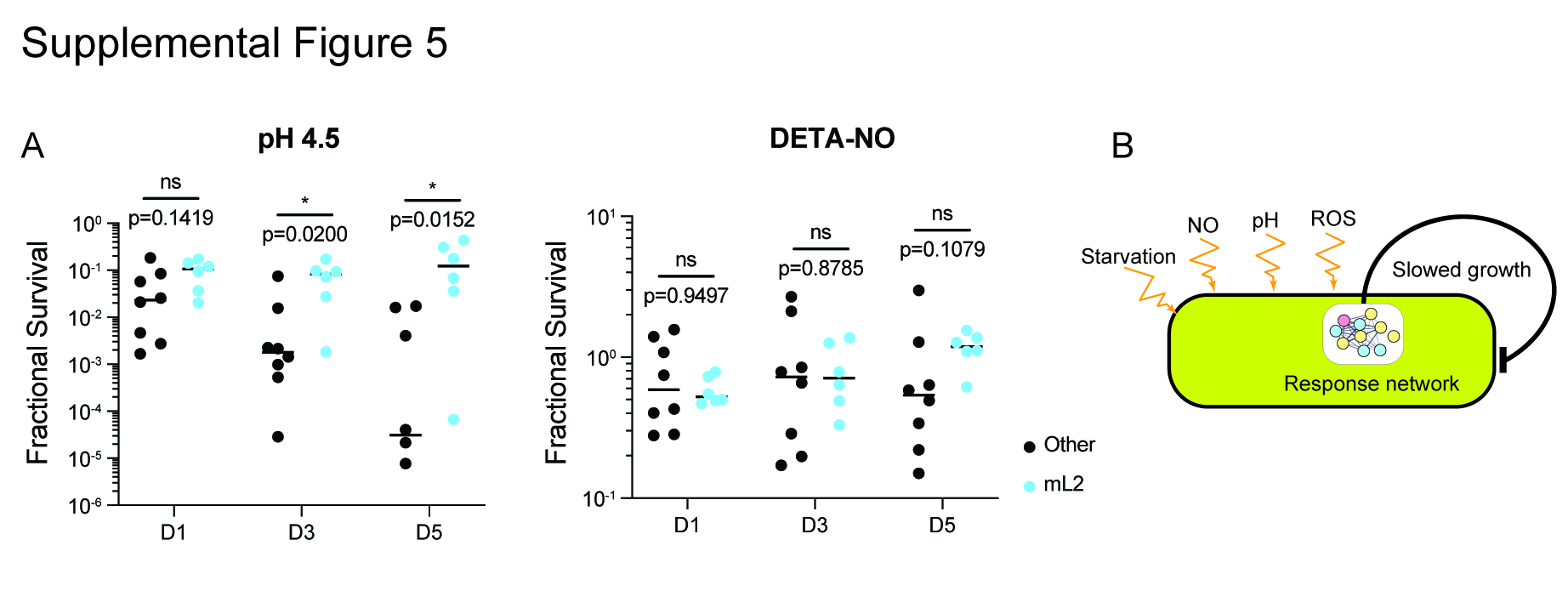

Supplement: FIG S5 [file msystems.00110-22-s0005.tif]
